# How manufacturing won or lost the COVID-19 vaccine race

**DOI:** 10.1016/j.vaccine.2023.12.031

**Published:** 2024-02-15

**Authors:** Michael L. King

**Affiliations:** Department of Chemical Engineering, University of Virginia, 351 McCormick Road, PO Box 400741, Charlottesville, VA 22904, United States

**Keywords:** COVID-19 vaccines, CMC, Manufacturing, Scale-up, Tech transfer, Quality by design

## Abstract

The development of vaccines for COVID-19 occurred at an unprecedented pace, and 32 vaccines using a broad range of technologies had received authorization for use on an emergency basis by the end of 2021, from either a national regulatory authority or the World Health Organization. However, 27 of those 32 vaccines had little impact on the global course of the pandemic. Only five vaccines, from AstraZeneca, Pfizer/BioNTech, Sinovac, Moderna, and Sinopharm, were manufactured, authorized, and distributed in time to significantly impact the number of deaths worldwide. Together, these five vaccines averted an estimated 17 million deaths in the first year of the vaccination campaign. The shared characteristic of these five manufacturers was their ability to rapidly develop and scale up vaccine production to deliver the large manufacturing volumes required to immunize large segments of the global population.

Because the development and manufacturing of these vaccines was generally on the critical path to authorization and supply, the technical activities involved with development, scale-up, testing, technology transfer, and full-scale manufacturing, as well as aspects of the Chemistry, Manufacturing, and Controls (CMC) regulatory interactions, are examined for each vaccine and technology for which information is available in the public domain to provide lessons learned and recommendations on proactive actions to better prepare us for a future pandemic response.

The critical success factors include prior experience with commercialization and approval, robust quality systems, rigorous process development strategies, flexible manufacturing facilities with a skilled workforce, collaboration, access to consumables, reagents, and adjuvants (if relevant), and an equitable distribution of the global vaccine manufacturing network.

## 1 Introduction

The pace and breadth of the global rollout of vaccines during the COVID-19 pandemic was unprecedented. Within two years, 369 vaccine candidates, covering a wide range of vaccine technologies, were in development [1], and many had been authorized for use in less than one year. By the end of 2021, a total of 32 vaccine products had received authorization for use on an emergency basis from one or more national regulatory authorities and/or the World Health Organization (WHO), and approximately 11 billion doses had been produced [2]. It is estimated that this impressive response prevented approximately 20 million deaths [3], [4]. However, global access to vaccines was not equitable [5] and, if the target set by the WHO of vaccinating 40 % of the population of every country by the end of 2021 had been achieved, an additional estimated 600,000 deaths could have been prevented [6].

This paper reflects on some lessons learned from the pandemic response with a focus on the Chemistry, Manufacturing, and Controls (CMC) aspects of vaccine development: what went well as well as opportunities for improvement in process development, scale up, technical transfers, workforce development, and manufacturing supply chains as part of a future pandemic response.

## 2 Approaches that worked well and contributed to overall success

### 2.1 Unprecedented speed of COVID-19 vaccine development

**Fig. 1:**
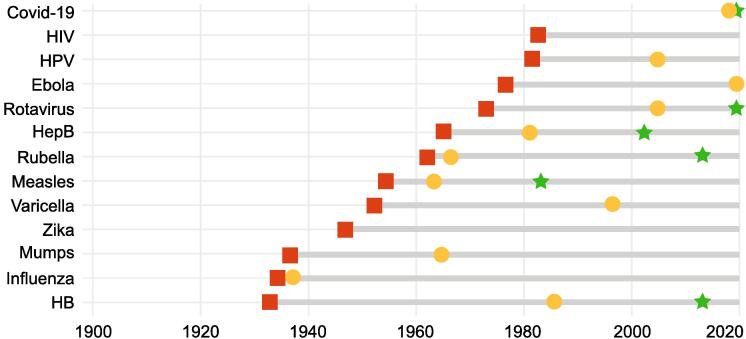
The time from identification of a microbe to substantial vaccination has continued to shorten (adapted from [8]).  Identification of microbe;  First vaccine developed;  40% vaccinated.

The speed of vaccine development and vaccine uptake for COVID-19 set new records. The new coronavirus, SARS-CoV-2, was isolated by January 7, 2020, and the genetic sequence was shared globally on January 12, 2020. While normal vaccine development can take a decade or more [7], by the end of 2020, no less than ten vaccines had received some level of authorization for use [2], [8]! Global vaccine uptake also set a record: although the time from vaccine development to widespread vaccination was already decreasing steadily, the remarkably short interval for COVID-19, measured in months rather than years, was unprecedented (Fig. 1) [8].

The rapid pace of COVID-19 vaccine development was achieved by leveraging recent technical advances as well as by investing at risk in process scale-up and manufacturing capacity, but without compromising patient safety. Numerous factors converged to allow such a rapid pace:

• The rapid identification of the SARS-CoV-2 virus and the selection of the spike protein as a key antigen target were made possible by previous work on zoonotic viruses such as Middle East Respiratory Syndrome (MERS) and severe acute respiratory syndrome (SARS).
• The design of prototype vaccines for early testing was accelerated by the pre-investment in several vaccine technology platforms over the last decades:
  o Messenger RNA (mRNA) and lipid nanoparticle (LNP) delivery systems
  o Non-replicating viral vectors
  o Protein subunits with adjuvants
• The rapid preparation of clinical and commercial supplies was facilitated by the utilization of existing flexible vaccine manufacturing capacity.
• The development timelines were compressed because greater financial risk was assumed by manufacturers, governments, and other funders such as Non-Governmental Organizations (NGOs) during the pandemic. Several approaches were implemented:
  o Parallel (rather than sequential) clinical development combined with large amounts of disease burden allowed more rapid statistical readout of clinical efficacy
  o Manufacturing processes were scaled up at risk (before Phase I data)
  o Clinical Phase 1/2 or Phase 2/3 studies were overlapped or combined
  o Commercial manufacturing was initiated at risk before the clinical efficacy was known
  o Greater regulatory flexibility allowed faster regulatory actions by utilizing rolling submissions, rapid reviews, and the use of emergency use authorizations before full approval
• Greater knowledge sharing resulted in dissemination of clinical and development data in real time. In February 2020, WHO, in collaboration with the Global Research Collaboration for Infectious Disease Preparedness (GloPID-R), hosted a meeting to share what was known about COVID-19 and identify a set of global research priorities [9]. In addition, preprint servers made manuscripts available in advance of peer review: in the first four months after the first confirmed case, over 16,000 articles were published, and 6,000 of those were first available as preprints hosted on BioRxiv or MedRxiv [10]. Furthermore, some common CMC issues were addressed in the workshops facilitated by COVAX CMC SWAT teams, as discussed in Section 3.2.
• And finally, a shared global sense of urgency, motivation, and hard work!

### 2.2 Diversity of vaccine technology platforms

**Fig. 2:**
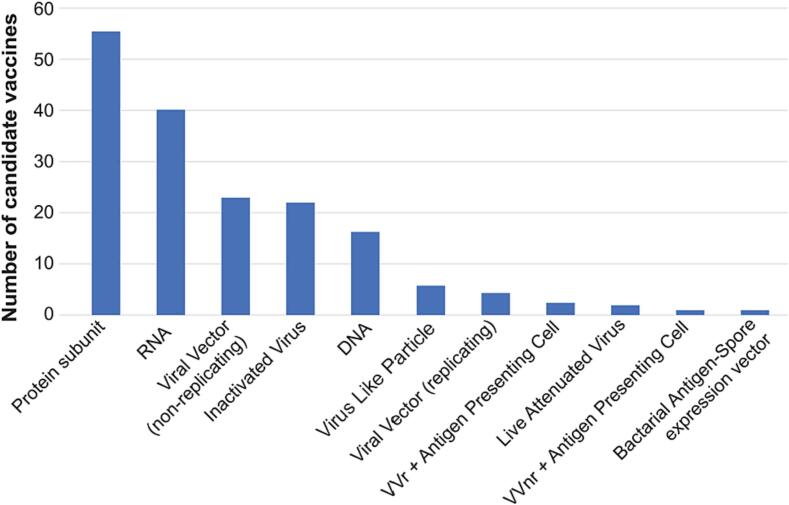
Vaccines in clinical development use a variety of technologies (adapted from [1],

The diversity of vaccine technology strategies was also noteworthy. Across the 369 programs, at least 11 different vaccine approaches have been explored (Fig. 2). By the end of 2021, a total of 32 products using five different technologies had received some form of authorization for use globally by at least one national regulatory agency (Table 1). By May 2022, the WHO had given Emergency Use Listing (EUL) to a total of 11 vaccines using four different technologies, which were approved as shown in Table 2 [11].

**Table 1:** Numerous Covid-19 vaccines developed using various technologies were authorized by at least one regulatory agency by the end of 2021 (adapted from [2], accessed January 2022).

**First Approval**		**Vaccine**	**Platform**
2020	Jul 22	Sinopharm (Wuhan) – Inactivated	Inactivated
	Jul 22	Sinopharm (Beijing) –BBIBP-CorV	Inactivated
	Jul 22	CanSino – Ad5-nCOV	Non-replicating viral vector
	Jul 22	Sinovac – CoronaVac	Inactivated
	Aug 11	Gamaleya – Sputnik V	Non-replicating viral vector
	Oct 13	Vector – EpiVacCorona	Protein subunit
	Dec 02	Pfizer BioNTech – COMIRNATY	mRNA
	Dec 18	Moderna – SPIKEVAX	mRNA
	Dec 20	Serum Institute of India – COVISHIELD	Non-replicating viral vector
	Dec 30	AstraZeneca – VAXZEVRIA	Non-replicating viral vector
2021	Jan 03	Bharat Biotech – COVAXIN	Inactivated
	Jan 15	Janssen – Ad26.COV2.S	Non-replicating viral vector
	Feb 19	Chumakov – Covi-Vac	Inactivated
	Mar 01	Anhui ZL – Recombinant SARS-CoV-2	Protein subunit
	Apr 22	RIBSP – QuazCovid	Inactivated
	May 06	Gamaleya –Sputnik Light	Non-replicating viral vector
	May 14	Biokantai (Beijing Minhai) – Inactivated	Inactivated
	Jun 09	Beijing Institute of Medical Biology – Inactivated	Inactivated
	Jun 14	Shifa Pharmed – Coviran Barekat	Inactivated
	Jun 29	Finlay – Soberana 2	Protein subunit
	Jul 09	CIGB – Abdala	Protein subunit
	Jul 19	Medigen – MVC-COV1901	Protein subunit
	Aug 20	Zydus – ZyCov-D	DNA
	Aug 20	Finlay – Soberana Plus	Protein subunit
	Sep 09	ODIR – FAKHRAVAC	Inactivated
	Oct 08	Vaxine/Cinnagen – SpikoGen	Protein subunit
	Nov 01	Serum Institute of India – COVAVAX	Protein subunit
	Nov 26	Gamaleya –Sputnik M	Non-replicating viral vector
	Dec 20	Novavax – NUVAXOVID	Protein subunit
	Dec 27	Erciyes University – Turkovac	Inactivated
	Dec 28	Sinopharm (NVSI) – Recombinant	Protein subunit
	Dec 29	Biological E – CORBEVAX	Protein subunit

**Table 2:** Vaccines using four technologies received WHO EUL by Sep 2022 (adapted from [11]).

**Technology**	**Vaccine**	**Manufacturer**	**First EUL**
**mRNA**	COMIRNATY	Pfizer/BioNTech	Dec 2020
	SPIKEVAX	Moderna	Apr 2021
**Non-replicating** **virus**	VAXZEVRIA	AstraZeneca	Feb 2021
	COVISHIELD	Serum Institute of India	Feb 2021
	Covid-19 Vaccine Ad26.COV2-S	Janssen	Mar 2021
	CONVIDECIA	CanSino Biologics	May 2022
**Inactivated**	Inactivated Covid-19 Vaccine (Vero Cell)	Beijing Inst. Biol. Products	May 2021
**viral vaccine**	CORONAVAC	Sinovac	Jun 2021
	COVAXIN	Bharat Biotech	Nov 2021
**Protein subunit** **with adjuvants**	COVOVAX	Serum Institute of India	Dec 2021
	NUVAXOVID	Novavax	Dec 2021

## 3 The role of CMC/process development and manufacturing supply in the pandemic response

Critical to such a rapid response was the development of robust vaccine production processes to support the clinical and commercial requirements of each of these vaccine technology platforms. Furthermore, in almost all cases, the CMC (Chemistry, Manufacturing and Controls) section of the regulatory application and the manufacturing supply were on the critical path to authorization and use in vaccinations. Multiple COVID-19 vaccine programs were reviewed for this paper, and some key enablers for robust CMC process development, scale up, and manufacturing were identified and will be described.

### 3.1 Examples of CMC/technical transfer experiences for drug substance technologies

The following discussion is based upon vaccine programs that achieved WHO emergency authorization (EUL) by Sept 2022 and for which sufficient public information was available. Several inactivated viral vaccines from China and India also achieved EUL by Sept 2022, but there was insufficient public data to include in the discussion.

#### 3.1.1 Messenger RNA (mRNA)

The promise of mRNA vaccines has been pursued for over two decades by a variety of companies and organizations (CureVac was founded in 2000, BioNTech in 2008, and Moderna in 2010). However, several challenges needed to be addressed to achieve clinical efficacy. These included the instability of the mRNA, the reactogenicity of the mRNA to the immune system, and the need for an effective formulation to deliver the mRNA to the targeted cells in the immune system. These challenges were overcome during the last few years with the use of modified nucleosides (replacing uridine with pseudo uridine) and the utilization of lipid nanoparticles (LNPs) to deliver the mRNA to the desired cells [12]. This is the approach that both Moderna and Pfizer/BioNTech took with their COVID-19 programs. This final formulation development was “just in time” for the pandemic response, and a highly effective vaccine was developed and approved in a remarkable 11 months!

**Pfizer and BioNTech** announced their partnership for a COVID-19 mRNA vaccine on March 17, 2020, just six days after the WHO declared COVID-19 a pandemic [13]. They utilized the existing capacity at Pfizer and BioNTech facilities as well as a network of contract manufacturing organizations (CMOs) to produce 3 billion doses of vaccine by the end of 2021 [14]. A key step was the preparation of LNPs, which are formed by impinging jet mixers. There was not enough time to scale up the process, so they scaled out by using a network of impinging jet mixers in parallel [15], [16]. This approach greatly reduced the risk of scaling up the LNP formulation step within the limited development timeline. Furthermore, since LNP technology was novel, a robust supply of the required lipids had not been developed to provide the quantity needed. Pfizer took the initiative to develop their own supply of the cationic lipid in-house at their Kalamazoo facility. They also had a very sophisticated global distribution capability for the ultracold temperatures required for storage of the final vaccine. This capability was deployed very effectively in highly developed areas and large cities, but the requirement for ultracold storage limited the ability to deliver in low resource settings and rural areas [17]. Pfizer’s commercialization experience (including the use of Quality by Design (QbD) and de-risking technical transfers by scaling out where appropriate) and knowhow with their “platform process” [18] proved invaluable to the rapid supply response. Pfizer also had a large, highly skilled workforce that could be re-directed to the COVID-19 vaccine project [16]. The vaccine received EUA from the FDA on Dec 11, 2020 and authorization from the EMA on Dec 21, 2020. Pfizer/BioNTech produced approximately 3 billion doses by the end of 2021 and had a major impact by preventing an estimated 6 million deaths [2], [14], [19].

**Moderna** did not have the same level of commercial experience as Pfizer, so they partnered with Lonza, Rovi, and Catalent, all well-established and experienced CMOs, to help with their commercial supply chain. The partners utilized a “modular” approach to capacity by adding “skids” of capacity for drug substance and formulation into drug product. This scale-out approach greatly reduced the risk in technical transfers and allowed the addition of units of capacity without disrupting the existing supply chain [20]. However, even with the experience of Lonza and the strategy of module scale-out, the rapid increase in production was challenged by the lack of a skilled workforce, and some EU deliveries were delayed [21], [22]. Nonetheless, Moderna was able to ship 807 million doses in 2021, and 25 % of those went to low- and middle-income countries [23]. The vaccine received EUA from the FDA on Dec 18, 2020 and authorization from the EMA on Jan 06, 2021. By the end of 2021, this vaccine had prevented an estimated 1.7 million deaths [19].

#### 3.1.2 Viral vectors

**Oxford/AstraZeneca** - Oxford University had been working with the non-replicating chimp adenovirus ChAdOx1 for emerging pathogens such as Middle East Respiratory Syndrome (MERS), Zika, and flu, and it was being developed as a platform technology when COVID-19 emerged. As soon as the genetic sequence for COVID-19 became available in January 2020, they began work on a COVID-19 vaccine and they had a candidate for development by March 2020. As an academic institution, they lacked significant infrastructure and facilities to support commercial development, supply, and distribution, and so they partnered with AstraZeneca on April 30, 2020, to develop and distribute the vaccine [24], [25].

Oxford’s pre-pandemic platform process for the ChAdOx1 virus used HEK 293 cells [26] and served as the basis for the commercial process. Before the agreement with AstraZeneca, technical transfer had been initiated to five sites in four countries, including Serum Institute of India, one of the world largest vaccine manufacturers. After the agreement, AstraZeneca assumed responsibility for manufacturing process optimization, validation, and network extension. The drug substance network was extremely large and included over 16 CMOs [27], which strained multiple technical transfers and process validation workloads and increased the overall risks in the supply chain. One of those risks was the production of drug substance at Emergent BioSolutions in Maryland. Because of significant quality issues that emerged in March 2021, AstraZeneca was pushed out of the Emergent facility by the US government, which then turned over its operation to Johnson & Johnson for the Janssen vaccine [28]. Another challenge was the low drug substance productivity from the Belgian Novasep plant (later purchased by Thermo-Fisher). However, despite multiple technical transfer and supply chain challenges (such as nationalism reducing exports from India and some other countries), the Oxford/AstraZeneca vaccine received authorization from the EMA on Jan 29, 2021, and played a significant global role in the pandemic with a total production of nearly 3 billion doses by the end of 2021, of which 500 million doses were delivered by COVAX. An analysis by Airfinity [19] suggests that this vaccine alone saved 6.3 million lives the first year.

**Janssen** – The Janssen division of Johnson and Johnson had developed an AdVac® viral vector technology, which was used for a licensed Ebola program (Zabdeno®) as well as for a vector for respiratory syncytial virus (RSV) and Zika. The AdVac® technology is based on a replication-incompetent adeno 26 viral vector grown in PER.C6® cells. Janssen announced their vaccine candidate (in collaboration with Beth Israel Deaconess Medical Center in Boston) on March 30, 2020. The supply chain [24] began to develop in the US with support from Operation Warp Speed. The drug substance would be manufactured by Emergent BioSolutions, and the drug product would be manufactured by Catalent in Indiana and Grand River Aseptic in Michigan. Johnson & Johnson also signed an agreement with Merck to produce drug substance in North Carolina and drug product in Pennsylvania. In Europe, the drug substance was produced in their Leiden facility, and the drug product was produced in Reig Jofre in Spain, Sanofi Pasteur in France, and IDT Biologika in Germany. The Leiden facility was inspected in Jan 2021, and the FDA authorized the vaccine for emergency use on Feb 27, 2021, making it the third vaccine approved in the US. Unfortunately, the quality problems at the Maryland Emergent BioSolutions facility resulted in cross contamination, which caused 75 million doses of vaccine to be discarded and shut down production for months [29]. These problems also resulted in the destruction of drug product made at Aspen Pharmacare in South Africa from drug substance manufactured at Emergent. In August 2020, Janssen announced an agreement with Biological E in India to mass produce the vaccine. However, this production was limited because of a lack of materials attributed to the implementation of the Defense Production Act in the US. Because of the problems at Emergent BioSolutions, Janssen had to reduce their production target from 1 billion to 500–600 million doses for 2021 [30]. Airfinity estimated that this vaccine saved 676,740 lives by the end of 2021 [19].

#### 3.1.3 Protein Subunit/Adjuvant

**Novavax** - The Novavax vaccine technology is based on a subunit antigen protein produced by infection of a genetically modified insect virus in moth (Sf9) cells. The purified protein antigen is then mixed with a proprietary adjuvant (Matrix-M) to create a recombinant, protein-based nanoparticle. The Matrix-M adjuvant is derived from saponins, which are naturally occurring compounds in the bark of the *Quillaja saponaria* tree found in Chile. This recombinant protein-based nanoparticle approach to a vaccine had previously been developed by Novavax for both flu and Ebola [31]. Once the genetic information on the SARS-CoV-2 spike protein was available, they rapidly developed a vaccine candidate for COVID-19 by April 08, 2020 [32], [33]. However, Novavax had significant challenges in that they had never brought a vaccine to market, had no commercial manufacturing plant, and had not previously received regulatory authorization for a vaccine.

At the time, Novavax was financially constrained due to the failure of their RSV vaccine in 2016. In a restructuring effort that year, they laid off 30 % of their skilled workforce [34] and then sold their only plant in 2019. To support COVID-19 vaccine development, they relied heavily on funding from both the US Operation Warp Speed and the Coalition for Epidemic Preparedness Innovations (CEPI) [35], [36]. Since they had no internal manufacturing capability, they used CMOs and members of the Developing Countries Vaccine Manufacturers Network (DCVMN) for their production. In the US, vaccine for clinical trials was initially produced by Emergent BioSolutions [33], but Novavax was displaced when the facility was needed for other Operation Warp Speed vaccines [28]. Because of requirements for local production in supply agreements, their supply chain became very complex. The drug substance for the European supply chain was expected to come from the Bohumil plant in the Czech Republic (which they purchased) and Biofabri in Spain. The US supply was expected to come from the CMO FujiFilm in both North Carolina and Texas. The Asian supply was expected to come from SK Biosciences in Korea, the Indian supply from Serum Institute of India, and the Japanese supply from Takeda [24]. The technical transfer of the drug substance process into so many diverse factories was very challenging and did not go as expected [37], [38]. Significant issues developed with yields and inconsistent purity of the drug substance, which varied in purity between the various facilities. These manufacturing problems may have contributed to delays to regulatory authorizations [38], [39], [40]. The WHO and EMA EUL was obtained on December 20, 2021 based upon the CMC data from the manufacturing process used at the Serum Institute of India.

On June 14, 2021, Novavax announced impressive clinical results regarding the PREVENT-10 trial, with overall 90 % vaccine efficacy [41]. A year later, on June 7, 2022, the FDA Advisory Committee recommended an EUA for NUVAXOVID, and full approval was granted on July 13, 2022 after review of additional manufacturing changes that had been submitted just prior to the Advisory Committee meeting. This FDA approval was noteworthy in that it was the first vaccine approved in the US from an Indian manufacturing facility [42]. The EMA authorized the vaccine on Dec 20, 2021 and the additional supply site of SK Biosciences in South Korea on July 7, 2022 [43].

As noted above, most of the delays in approval experienced by NOVAVAX were reportedly due to manufacturing, CMC, and quality issues [37], [38], [40], [44]. Eventually, the process established at Serum Institute of India was used to gain regulatory approval for WHO, FDA, and EMA [42].

With all its challenges, Novavax was still the only protein subunit/adjuvant vaccine technology to gain WHO approval by October 2022. Several other protein subunit/adjuvant programs have had some local country approvals (e.g., Bio E/Dynavax with CORBEVAX, SK Bioscience/GSK with GBP510) but not WHO EUL.

### 3.2 CMC collaboration in the global pandemic response

Behind the scenes, there was extensive collaboration between organizations working to solve common problems encountered by vaccine developers. One key organization was the Covid-19 Vaccines Global Access (COVAX) facility established by the WHO in April 2020 [45], [46]. This global initiative was aimed at equitable access to COVID-19 vaccines and was directed by the Global Alliance for Vaccine Immunization (GAVI), CEPI, and the WHO, alongside key delivery partner UNICEF.

Under the COVAX organization, several workgroups were established, including the Manufacturing “SWAT” team, which provided cross-cutting support to COVID-19 developers on CMC issues [47]. The Manufacturing SWAT team was staffed with members of CEPI, WHO, UNICEF, US National Institutes of Health (NIH), GAVI, Bill and Melinda Gates Foundation, Program for Appropriate Technology in Health (PATH), International Federation of Pharmaceutical Manufacturers & Associations (IFPMA), Clinton Health Access Initiative (CHAI), DCVMN, regulatory agencies, and others.

One of the key approaches to addressing these cross-cutting issues was a series of virtual workshops with vaccine developers along with subject matter experts from industry, regulatory agencies, and non-governmental organizations. Each workshop focused on a set of specific issues common to all developers, and each was attended by hundreds of representatives from both large and small vaccine developers. Some of the COVAX Manufacturing SWAT team workshops are listed below, along with some of their outcomes [47]:

• Drug product (both initial and follow up meetings) – Created alignment with the need to move from single-dose to multi-dose vials to conserve scarce pharmaceutical glass vials and capacity in existing fill/finish facilities.
• Best practices for post-approval changes – Established strategies to address demonstration of product comparability for manufacturing changes and technical transfer to multiple manufacturing sites.
• Best practices for tech transfer – Reviewed best practices for technical transfer, including topics such as raw materials, consumables, documentation, teamwork, leveraging prior knowledge, and regulatory strategy.
• Accelerated vaccine development and experiences with regulatory pathways for vaccines in emergency situations.
• CMC comparability workshop – Provided a baseline understanding of issues regarding comparability as applied to the case of the ultra-rapid development and supply of COVID-19 vaccines.
• Best practices for determining and updating storage temperature and shelf life – This included the use of kinetic modeling to predict product stability and the addition of bar and QR codes to allow updates on product expiry dates. Unified barcoding & contribution to labeling/counterfeit countermeasures were also discussed.
• WHO presentation by WHO-National Control Laboratory Network for Biologicals.
• CMC implications of multivalent COVID-19 vaccines.

In addition to the Manufacturing SWAT team workshops, there were several other collaborative initiatives. One initiative was chartered in April 2020 to perform a “Manufacturing Capacity Landscape” analysis to determine the global capacity for vaccine production [48]. This initiative was undertaken by CEPI, BMGF, and CHAI and helped reveal the available manufacturing capacity for various drug substance technologies as well as for filling of vials and syringes for drug product. Where excess capacity was identified, it was offered by CEPI, acting as a “matchmaker”, to companies in need of additional capacity [49]. In addition, CEPI established the “COVAX Marketplace” where companies in need of raw materials or consumables could find and exchange goods [50].

### 3.3 Lessons Learned: Pandemic-specific factors challenging the vaccine supply

Several factors made the CMC, technical transfers, and supply chain issues extremely challenging during the pandemic. In addition to the technical complexity and rapid pace of development and manufacturing, both nationalism and capitalism contributed to disruptions in worldwide supply chains.

Nationalism affected both the CMC activities and the supply chain. Because many countries required manufacturing in their own country or region, additional regional manufacturing facilities and technical transfers were required [24]. In addition, the vaccine supply was initially scarce, and export bans from some countries were established to secure local availability for the country or region. This was true in almost all global regions (North America, South America, Europe, India/Asia). Governments were focused more on the acquisition of vaccine supply for their constituents and less on global equity in vaccine distribution [51]. For regions without vaccine manufacturing resources, such as Africa, the result was extremely limited supplies of vaccine [5].

Another driver of the complexity of supply chains was capitalism. Companies with successful vaccines were naturally incentivized to get the best price for their product. Although Pfizer, Moderna, and others sold vaccine at lower prices to GAVI/UNICEF for low- and middle-income countries [2], [16], they still generated significant revenue. In contrast, AstraZeneca’s “no-profit” pledge, which it made in Oct 2020 and maintained until Nov 2021, resulted in lower revenue. For example, in 3Q2021, AstraZeneca had $1 billion in revenue from their COVID-19 vaccine, whereas Moderna generated $5 billion and Pfizer/BioNTech generated $13 billion in revenues [52].

More broadly, for companies to negotiate a premium price as part of the supply contract agreements with various governments, there was often a requirement for local manufacturing. This further challenged developers with additional technical transfers to local manufacturers (often CMOs) and therefore additional risk. The high-income countries locked up most of the supply, leaving little for low- and middle-income countries. The WHO/COVAX tried to address this by focusing on rapid vaccination of the most vulnerable 20 % of the global population (older people and health care workers), but they rapidly fell short of this goal because of the lack of vaccine supply [3], [45], [51]. And for countries that did not possess local vaccine manufacturing infrastructure, such as many of those on the continent of Africa, that meant no local supply and total reliance on supply from other countries.

**Fig. 3:**
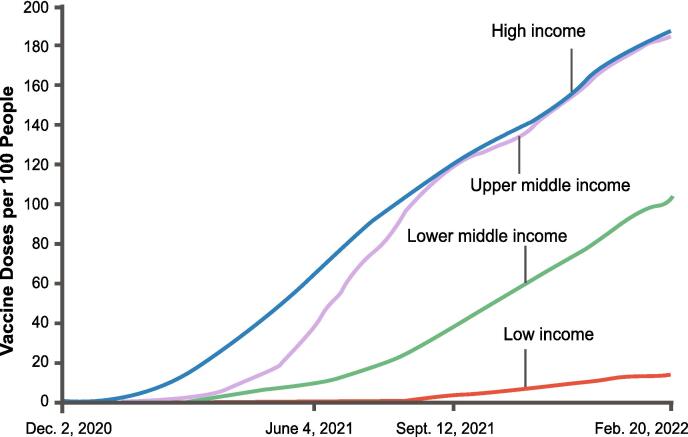
More COVID-19 vaccine doses were administered in higher income countries than in lower income countries, Dec 2, 2020 through Feb 20, 2022 [53], [54]. The income categories are defined by the World Bank, and the y-axis represents individual doses.

These factors led to a huge inequity in vaccine distribution. By Feb 2022, several high-income countries exceeded 90 % vaccine coverage, but only about 11 % of all people in low-income countries had received at least one dose (Fig. 3) [53], [54].

The COVID-19 response laid bare the unequal global distribution of the world’s vaccine manufacturing capacity and capabilities. The great majority of the manufacturing capacity was in high-income countries (North America and Europe) and middle-income countries (India, China, and South America). Low-income countries had almost no or very limited regional manufacturing capability. When this absence of manufacturing capacity was combined with the forces of nationalism and capitalism, the result was limited access to vaccine supplies. For example, in 2020, Africa could only manufacture 1 % of their vaccine supply. For equity in access, a more balanced global vaccine manufacturing network is desired [55].

Recently there have been significant efforts to address equitable geographical manufacturing. One example is the African Vaccine Manufacturing Initiative (AVMI) [56], which has signed up 10 manufacturers. The Partnership for African Vaccine Manufacturing (PAVM) under the African CDC is another significant effort in Africa [57]. In addition, several multinational corporations are building facilities in Africa, for example BioNTech in Rwanda and Moderna in Kenya; WHO is investing in an mRNA hub at Afrigen in Cape Town, South Africa; and significant investment has been made in the Institute Pasteur Dakar, Senegal by BMGF, CEPI, Wellcome Trust and others.

## 4 Applying lessons learned to future vaccines: Proactively addressing CMC process development issues

A review of what worked well for the COVID-19 vaccines along with opportunities for further improvement led to the following **key concepts and approaches that remain critical to the rapid development, scale-up, and manufacturing of new vaccines against any diseases**: **vaccine development and manufacturing experience** – Developers with a history of successful commercialization and registration of vaccines had a greater probability of success. Inexperienced developers and academic institutions fared better when they teamed up with a more experienced developer or CMO. Vaccines are very complex to manufacture and require both tacit, hands-on knowledge as well as explicit knowledge [58].

• **Robust quality systems** – Developers and CMOs with robust quality systems did much better in approvals and pre-inspection activities by both WHO and stringent regulatory authorities. Programs or companies that used facilities without sound quality systems suffered significant delays and disruption of supply chains [28], [29].
• **Rigorous process development strategies** – Developers that had a clear “line of sight” strategy from initial vaccine development to clinical production processes to the final commercial process performed better. Hand-offs were minimized, technical transfers were de-risked, and product risks were reduced for the full development process. Developers that utilized the concept of Quality by Design (QbD) [59] also fared much better. They understood the Critical Quality Attributes (CQAs) of the final product and determined how to consistently produce quality product by controlling the Critical Process Parameters (CPPs) that affect those CQAs. They also benefited from developing and implementing sound process analytical methods so that process performance could be effectively monitored and adjusted.
• **Flexible manufacturing facilities and skilled workforce** – During the pandemic, there was not enough time to build capacity via traditional greenfield stick-built construction. Instead, existing factories from vaccine developers, CMOs, and members of the DCVMN were modified and adapted to accommodate the COVID-19 vaccines. Factories with more open “ballroom” suites had the advantage of rapid assembly of bioreactors and purification equipment. Use of single-use equipment also streamlined the qualification timeline prior to production. Where additional capacity was needed, the use of modular approaches (such as G-CON cleanrooms, which were used by Pfizer [18]) greatly reduced the construction and validation timelines [60]. In addition to the factory facilities, it was essential for the manufacturers to have a highly skilled workforce readily available to operate the factories. A more equitable global distribution of facilities and skilled workforce is also desired.
• **Collaboration/knowledge sharing** – Mechanisms and opportunities (such as the COVAX SWAT Team workshops) that were established to promote effective sharing of best practices and resolve common issues in COVID-19 vaccine development (e.g., final multidose image, stability modeling, labeling/bar coding, technical transfer strategy, post-approval changes) should be considered in any global rapid pandemic response.
• **Access to adjuvants** - Protein subunit vaccines often require adjuvants to enhance vaccine efficacy.
  To increase the probability of success, especially when speed is critical, most developers prefer to choose among adjuvants that have previously been used in licensed vaccines, i.e., vaccines that demonstrated sufficient efficacy and safety to receive regulatory approval. However, this represents a very short list. For example, the US CDC lists only six adjuvants that have been used in vaccines approved for use in the US, and five of these are proprietary [61]. During COVID-19 vaccine development, access to these five adjuvants was limited and required companies to enter into collaboration agreements such as the one between GSK and Sanofi, where Sanofi contributed the antigen and GSK contributed the proprietary adjuvant [62]. To facilitate future development of protein-based vaccines, a more robust portfolio of accessible, low-cost, sustainable adjuvants is desired.

• **Access to consumables and reagents** - The huge ramp-up of vaccine production on top of current vaccine production severely stressed the manufacturing supply chains. A more strategic reserve of these materials might be desired.

## 5 Opportunities for a faster and more robust CMC pandemic response

The speed of the COVID-19 pandemic response, resulting in highly effective vaccines authorized for use in less than a year, was unprecedented! It has likely changed vaccine development timelines forever. In March 2022, CEPI published their proposal for a 100-day “moonshot” to deliver pandemic vaccines within 100 days [63]. Their analysis showed that, with current practices, the theoretical shortest possible period was 250 days. They went on to develop strategies to reduce that time to 100 days. That 100-day goal has been further supported by a report from the G7 in their “100 Days Mission to Respond to Future Pandemic Threats” [64].

One of the keys to achieving such an ambitious goal is to streamline the CMC and manufacturing activities to minimize the critical path. One recommendation is pre-investment into vaccine process technology platforms to facilitate the support of producing both clinical trial materials and commercial supply. This approach requires a clear “line of sight” during process development from product definition to final commercial manufacturing. In turn, such streamlining will require pre-investment into the development of process platforms to support each of the various vaccine technology platforms (e.g., mRNA, viral vectors, subunit proteins). Note that multiple vaccine platforms are required for the wide variety of potential pathogen targets – mRNA cannot address all pathogens since it is specific for protein antigens.

## 6 Conclusion

The global vaccine development response to the COVID-19 pandemic was unprecedented and resulted in a huge reduction in human deaths and illnesses. By reflecting on lessons learned, we can identify the critical success factors and develop recommendations and best practices to better prepare us for the next epidemic or pandemic and to achieve a better and more equitable global response.

## Funding

The publication of this paper was supported by the 10.13039/100000865Bill and Melinda Gates Foundation, 10.13039/100021038Seattle, WA [Grant numbers INV-050046 and INV-031633].

## Declaration of competing interest

The authors declare the following financial interests/personal relationships which may be considered as potential competing interests: Consultant for BMGF, Shares of stock in Merck (USA), Bristol Myers Squibb, and Moderna. Coalition of Epidemic Preparedness (CEPI) – Vice Chair of Scientific Advisory Committee and member of Portfolio Strategy Management Board. Global Alliance for Vaccine Immunization (GAVI) – Observer Independent Product Group.

## Data Availability

All information is in public domain as described in references
